# High species diversity of trichostrongyle parasite communities within and between Western Canadian commercial and conservation bison herds revealed by nemabiome metabarcoding

**DOI:** 10.1186/s13071-018-2880-y

**Published:** 2018-05-15

**Authors:** Russell W. Avramenko, Ana Bras, Elizabeth M. Redman, Murray R. Woodbury, Brent Wagner, Todd Shury, Stefano Liccioli, M. Claire Windeyer, John S. Gilleard

**Affiliations:** 1 Department of Comparative Biology and Experimental Medicine, University of Calgary Faculty of Veterinary Medicine, Calgary, Alberta Canada; 2 Department of Production Animal Health, University of Calgary Faculty of Veterinary Medicine, Calgary, Alberta Canada; 3 Present address: Feedlot Health Management Services Ltd., Okotoks, Alberta Canada; 4 Department of Large Animal Clinical Sciences, Western College of Veterinary Medicine, University of Saskatchewan, Saskatoon, Saskatchewan Canada; 5 Department of Veterinary Microbiology, Western College of Veterinary Medicine, University of Saskatchewan, Saskatoon, Saskatchewan Canada; 6 Department of Veterinary Pathology, Western College of Veterinary Medicine, University of Saskatchewan, Saskatoon, Saskatchewan Canada; 7 Grasslands National Park, Parks Canada, Val Marie, Saskatchewan Canada

**Keywords:** Bison, Nemabiome, Metabarcoding, *Ostertagia ostertagi*, *Haemonchus placei*, *Cooperia oncophora*, Nematodes

## Abstract

**Background:**

Many trichostrongylid nematode species are reported to infect bison, some of which are major causes of disase and production loss in North American bison herds. However, there is little information on the species distribution and relative abundance of these parasites in either commercial or conservation herds. This is largely because trichostrongylid nematode species cannot be distinguished by visual microscopic examination of eggs present in feces. Consequently, we have applied ITS2 rDNA nemabiome metabarcoding to describe the trichostrongyle parasite species diversity in 58 bison production groups derived from 38 commercial North American plains bison (*Bison bison bison*) herds from across western Canada, and two bison conservation herds located in Elk Island National Park (EINP) [plains bison and wood bison (*Bison bison athabascae*)] and one in Grasslands National Park (GNP) (plains bison).

**Results:**

We report much higher infection intensities and parasite species diversity in commercial bison herds than previously reported in beef cattle herds grazing similar latitudes. Predominant trichostrongyle parasite species in western Canadian commercial bison herds are those commonly associated with Canadian cattle, with *Ostertagia ostertagi* being the most abundant followed by *Cooperia oncophora*. Combined with high fecal egg counts in many herds, this is consistent with significant clinical and production-limiting gastrointestinal parasitism in western Canadian bison herds. However, *Haemonchus placei* was the most abundant species in five of the production groups. This is both surprising and important, as this highly pathogenic blood-feeding parasite has not been reported at such abundance, in any livestock species, at such northerly latitudes. The presence of *Trichostrongylus axei* as the most abundant parasite in four herds is also unusual, relative to cattle. There were striking differences in parasite communities between the EINP and commercial bison herds. Most notably, *Orloffia bisonis* was the predominant species in the wood bison herd despite being found at only low levels in all other herds surveyed.

**Conclusions:**

This study represents the most comprehensive description of parasite communities in North American bison to date and illustrates the power of deep amplicon sequencing as a tool to study species diversity in gastrointestinal nematode communities.

**Electronic supplementary material:**

The online version of this article (10.1186/s13071-018-2880-y) contains supplementary material, which is available to authorized users.

## Background

The North American bison industry currently comprises almost 500,000 farmed North American plains bison distributed across approximately 4000 farms [1, 2]. In contrast, less than 20,000 bison (~4% of the total population) are managed for conservation purposes [3] across 62 conservation herds, the majority of which are small (i.e. < 400 animals) [2].

Demand for bison meat in the USA has increased 26% over the last three years, and 360 million US dollars were spent on bison meat products in 2016 [4]. Bison meat is marketed as a natural food, raised without the use of antimicrobial drugs, and high in protein, low in total fat, with a favorable omega fatty acid profile [5]. Gastrointestinal nematode parasites, particularly the trichostrongylid group, are one of the most important causes of disease and production loss in commercial bison [6]. There is convincing anecdotal evidence from practicing veterinarians that the clinical and production impacts of gastrointestinal parasitism are more severe in bison than those seen in beef cattle under similar climate and grazing situations. Clinical disease and visible effects on body condition are not uncommon, with *Ostertagia ostertagi* being considered the most pathogenic species overall. Clinical case reports describe fatalities and classic parasitic gastroenteritis signs such as diarrhea, emaciation and anemia [7, 8]. As parasites can have detrimental effects on host survival and lead to trade-offs between reproductive effort and parasite resistance in wild ungulate populations [9, 10], parasitism has implications also for management of bison free-ranging and conservation herds.

There are many different trichostrongylid nematode species reported to infect bison in North America based upon necropsy work and clinical case reports. These include *Ostertagia ostertagi*, *Orloffia bisonis*, *Haemonchus contortus*, *Haemonchus placei*, *Trichostrongylus axei*, *Trichostrongylus longispicularis* (syn. *T. lerouxi*), *Cooperia oncophora*, *Cooperia surnabada, Nematodirus helvetianus*, *Oesophagostomum radiatum* and *Chabertia ovina* [8, 11, 12]. The majority of these species have visually indistinguishable eggs, making their differentiation to the species level very difficult. Culturing of feces to harvest and morphologically identify third-stage larvae (L3) is a time-consuming, skilled task and because it generally only determines identity to the genus level, is rarely done. Similarly, molecular identification has only rarely been applied to bison parasites and scalable quantitative molecular assays have not yet been developed [8]. Consequently, the relative prevalence of each species in different geographical regions, types of herd, and production systems, is largely unknown. As different trichostrongylid nematode species vary in terms of clinical signs and pathogenicity potential for their hosts, addressing such a knowledge gap is relevant for both the commercial bison industry and conservation.

We have recently developed a deep amplicon ITS2 rDNA sequencing approach, termed “nemabiome” metabarcoding, for identification of strongylid L3 larvae cultures from cattle feces [13, 14]. This allows accurate relative quantification of strongylid nematode species in fecal samples on a much larger scale than previously possible. In this paper, we illustrate the value of this approach in a survey of 58 North American plains bison (*Bison bison bison*) production groups from 38 commercial herds across western Canada, as well as three national park conservation bison herds located in Canadian national parks: 2 plains bison (*Bison bison bison*) and 1 wood bison (*Bison bison athabascae*). The data presented provide the most comprehensive insights into nematode parasite species distribution in bison herds to date and reveal a high level of trichostrongylid nematode species diversity both within and between Canadian bison herds.

## Methods

### Fecal collection and parasitological analysis

**Fig. 1:**
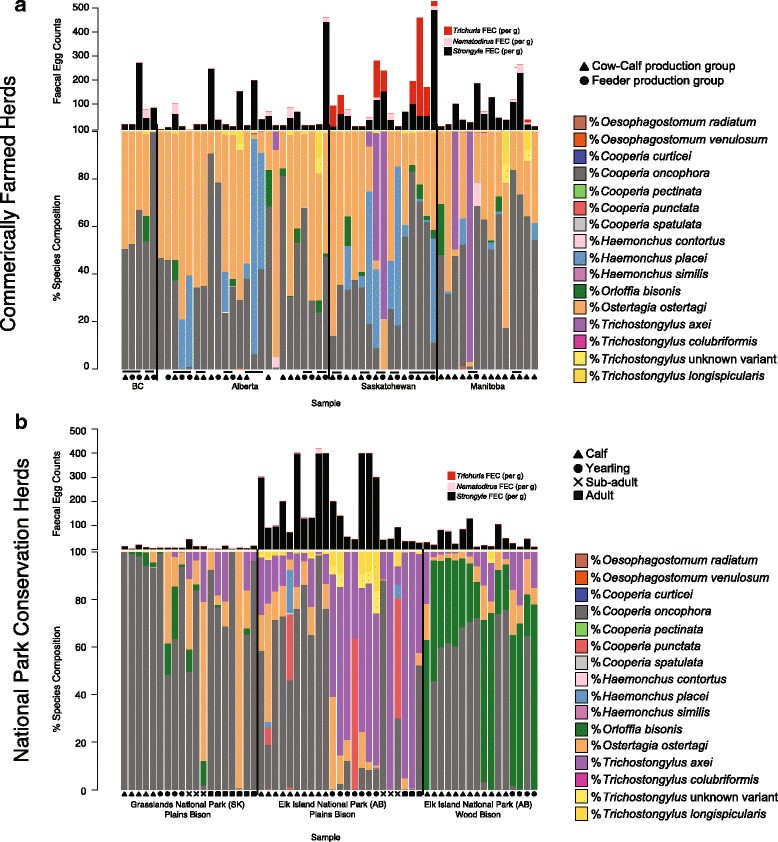
The proportions of parasitic nematode species from commercial and conservation bison herds are shown. **a** Fecal samples were collected from 58 bison production groups from 38 commercial herds from across western Canada: BC (*n* = 5); AB (*n* = 24); SK (*n* = 15); MB (*n* = 14). Each stacked bar-chart represents the species composition of a production group as determined by nemabiome sequencing. 10–20 fecal pats were sampled and pooled together from each production group within a herd. Two fecal egg counts (FEC) were performed on each pool, counting strongyle, *Nematodirus* and *Trichuris* eggs. The mean of these egg counts is displayed above each bar chart. Triangles represent cow-calf production groups; circles represent feeder production groups. Lines indicate that the groups belong to the same herd, and are likely to have similar management protocols and have close geographical proximity to one another. **b** Fecal samples were collected from individual bison from three conservation bison herds found within Canadian national parks, each stacked bar-chart represent the species composition of an individual animal sampled: GNP, plains bison herd (*n* = 19); EINP, plains bison herd (*n* = 23); EINP, wood bison herd (*n* = 16). Two fecal egg counts (FECs) were performed on each pool, counting strongyle, *Nematodirus* and *Trichuris* eggs. The mean of these egg counts is displayed above each bar chart. Animal age is indicated by a triangle (calves), circle (yearlings), ‘X’ (sub-adults) or square (adults)

Fecal samples were collected from 58 bison production groups from 38 herds across western Canada [British Columbia (*n* = 5), Alberta (*n* = 24), Saskatchewan (*n* = 15), Manitoba (*n* = 14)] between October 14, 2014 and January 16, 2015, in accordance with an approved Animal Use Protocol (Animal Care Committee, Study #AC13-0157, University of Calgary). The commercial herds were originally described in Bras et al. [15], who provide detailed descriptors in regards to how the herds were selected. In this paper, a ‘herd’ is operationally defined as all bison production groups managed by a single producer. Between 10 and 20 individual, freshly voided fecal pats (corresponding to ~4–8% of the herd on average) were sampled and pooled from the pasture of each of the main production groups within a herd during farm visits. The production groups were defined as: cow-calf groups (mature cows and bulls, replacement heifers and calves) or feeder groups (yearling bison being fed for slaughter). Pooled samples from each production group were kept separate, resulting in between 1 and 4 groups being sampled per herd (Fig. 1).

Fecal samples were also collected from three different family groups, each in distinct bison herds in two Canadian national parks: the Grasslands National Park (GNP, Saskatchewan) plains bison herd (19 samples), the Elk Island National Park (EINP, Alberta) wood bison herd (16 samples) and the EINP plains bison herd (23 samples). Fecal samples were collected *per rectum* in a squeeze chute during round-up activities, when individuals were differentiated in age groups based on tooth eruption and wear. All fecal samples were placed in separate plastic bags with the air expelled and shipped with cool packs.

Following homogenization of each pooled sample from commercial bison herds, fecal egg counts (FECs) were performed using the Paracount-EPG 3-chamber McMaster Fecal Egg Count Test following the manufacturers recommended procedure. FECs from national park bison samples were performed using a modified Wisconsin method [16]. Eggs of strongyle, *Nematodirus*, *Trichurius*, *Moniezia*, *Capillaria*, *Toxocara* and *Strongyloides* and oocysts of *Eimeria* were identified and counted separately. Two FECs were performed on each pooled sample and the mean of the two counts used for data analysis. In order to determine the species composition of the strongyle nematode communities, coprocultures were set up with the remaining feces as described by Roberts & O’Sullivan [17], but with the use of vermiculite rather than sawdust and incubated at room temperature (~21 °C) for 21 days. The L3 larvae were harvested and stored in tap water at 6 °C. Only strongyle species develop to L3 during coproculture. Strongyle L3 larvae were harvested from each sample into aliquots of 1000–2000 larvae dependent on the total number of larvae obtained, fixed in 70% ethanol, and stored at -80 °C until needed.

### Deep-amplicon sequencing of ITS2 rDNA region

Bulk DNA lysates were prepared from larval populations, and the ITS2 rDNA fragment was PCR-amplified and sequenced by deep amplicon sequencing as previously described and validated for the nemabiome metabarcoding assay [13]. Sequencing was completed on the Illumina MiSeq with the 2× 250 v2 Reagent Kit. An average read depth of ~19,000 was obtained for each sample. Samples were removed if they did not have at least 2000 reads, as this was taken to indicate suboptimal PCR amplification.

### Sanger sequencing ITS2 derived from individual L3 larvae

Individual larvae were picked, lysed, PCR amplified and sequenced as previously described in Avramenko et al. [13].

### Bioinformatic analysis

Samples were analyzed using the Mothur bioinformatic tool version 1.36.1 [18]. Raw pair-end reads were assembled creating single contigs. Reads were filtered and removed if they were < 200 bp or > 450 bp or if there were ambiguities between the overlapping reads. A database containing ITS2 rDNA sequences from all relevant nematodes was created from public databases and can be found in Additional file 1: FASTA Database. Reads were aligned to the ITS2 rDNA database and were discarded if they did not align to at least 10% of an ITS2 rDNA amplicon with at least 90% sequence similarity. Remaining sequences were classified as corresponding to reference sequences in the database, using the *k*-nearest-neighbor method with *k* = 3. Sequences in which the three nearest matches were not a single species were then assessed at the next taxonomic level until a consensus could be reached. Read counts were adjusted to account for inherent assay biases that could include species-specific differences in DNA content, DNA lysis efficiency, ITS2 amplification, or copy number as previously described in Avramenko et al. [13]. Read counts assigned to each species were multiplied using previously validated correction factors: *C*. *oncophora* = 1.183576925; *T*. *axei* = 1.296071418; *T*. *colubriformis* = 0.584257283; *C*. *punctata* = 2.779407405; *O*. *ostertagi* = 1.294841767; and *H*. *placei* = 0.919208106. Percentage species composition of each sample was calculated by dividing the total reads assigned to each species by the total number of reads per sample to obtain a relative percentage of each species. Species with a prevalence below 0.05% were removed, as this translates to less than 1 worm per sample and is thus likely to be contamination or misidentification of reads [13]. Sequences identified as *Nematodirus* were removed from the analysis, as the coproculture technique does not allow for the suitable culturing of the species. Since *Nematodirus* eggs are visually distinguishable, estimations of *Nematodirus* intensities were provided by the observed egg counts.

### Statistical analysis

Descriptive statistics including mean, standard deviation, standard error and 95% confidence intervals were calculated using the *Descriptives* function of SPSS Statistics (IBM Corp. Released 2016. IBM SPSS Statistics for Macintosh, Version 24.0. Armonk, NY: IBM Corp). To assess differences in strongyle, *Nematodirus* and *Trichuris* egg counts between populations, a one-way ANOVA, assuming non-equal variances using a Games-Howell *post-hoc* test, was performed using SPSS Statistics.

Alpha diversity of each assessed sample was calculated through the inverse Simpson index [19]. The calculations were performed in Mothur v.1.36.1 using the built-in inverse Simpson calculation. All samples were subsampled to 2000 reads to ensure equal comparisons. Samples with fewer than 2000 reads were removed from analysis. To assess whether the inverse Simpson index differed significantly between different populations, a one-way ANOVA, assuming non-equal variances and using a Games-Howell *post-hoc* test, was performed using SPSS Statistics.

Diversity was additionally measured with an AMOVA (Analysis of Molecular Variance), using the calculator built into Mothur v.1.36.1. The AMOVA calculations were performed with alpha set to 0.05 and 1000 iterations. To assesses the level of dissimilarly in composition between defined groups and populations, beta diversity (ranging from 0 for complete identity to 1 for complete diversity between the populations) was calculated using the weighted Bray-Curtis dissimilarity index, performed in Mothur v.1.36.1 using the weighted-UniFrac function [20, 21].

To identify which features (i.e. species) are differentially abundant between two populations, beta diversity estimations between populations on a species and genera basis were accomplished with the MetaStats plugin in Mothur v. 1.36.1, using 1000 permutations and default parameters [22]. As MetaStats assumes the non-normal distribution of data, it runs a modified nonparametric t-test to assign a *P*-value of significance [22]. As this method can overestimate the significance of lowly abundant features, statistical differences were not considered if the species were present at less than 2% on average in one of the comparable groups.

The bar charts displayed in figures were created in R Studio version 0.99.489.

### Sequence analysis and phylogenetic trees

For the interrogation of unclassified sequences, consensus sequences representing all parasite species included in the analysis were created. A list of all sequences and accession numbers used are provided in Additional file 1: FASTA Database. For each species, all available sequences were aligned using Geneious (version 10.1.3 (created by Biomatters), available from http://geneious.com/), using the MUSCLE algorithm with default parameters. A consensus sequence was generated for each species, from each alignment, with a 75% threshold.

Unclassified *Trichostrongylus* sequences identified during the species analysis were extracted from all samples using the “*get.lineage*” command in Mothur. These sequences were visualized in Geneious, and aligned using MAFFT, with the FFT-NS-1 algorithm. Once aligned, the file was split according to the dominate sequence variant observed visually. The variant file was then re-aligned, and a consensus sequence generated using a 75% threshold.

All consensus sequences were then aligned in Geneious using MUSCLE, with default parameters and a phylogenetic tree was computed with the MrBayes plugin with the GTR substitution model, with 500,000 chain length and rooted on the *Trichuris discolor* consensus sequence.

## Results

### Parasite herd-level prevalence and infection intensities in commercial and conservation bison herds

Fecal egg/oocyst counts for strongyle, *Nematodirus*, *Trichuris*, *Eimeria*, *Moniezia*, *Capillaria*, *Toxocara* and *Strongyloides* were determined for 58 commercial bison production groups from 38 herds and three national park conservation herds (fecal samples from individuals in each herd). The summary statistics for the fecal egg count data are shown in Tables 1 and 2, and Fig. 1a, b (breakdown of FECs by province can be viewed in Additional file 2: Table S1).

**Table 1:** Fecal egg counts for commercial bison herd production groups (58 groups sampled)

Parasite	No. of positive production groups	EPG ± SD (range)	Prevalence (%)
Strongyle	57	72.0 ± 99.6 (0–493)	98.3
*Nematodirus*	15	5.1 ± 11.0 (0–42.5)	25.9
*Trichuris*	9	18.0 ± 61.4 (0–408)	15.5
*Moniezia*	11	20.7 ± 57.9 (0–306)	19.0
*Capillaria*	1	0.7 ± 4.8 (0–34)	1.7
*Strongyloides*	0	na	0
*Toxocara*	0	na	0
*Eimeria*	49	656.7 ± 1124.2 (0–7174.0)	84.5

*Abbreviation*: *na* not applicable

**Table 2:** Fecal egg counts for conservation bison herds

Herd (*n)*	Parasite	No. of positive individuals	EPG ± SD (range)	Prevalence (%)
GNP plains bison herd (*n* = 19)	Strongyle	19	11.2 ± 10.1 (2–42.0)	100
	*Nematodirus*	4	0.04 ± 0.08 (0–0.2)	21.0
	*Trichuris*	1	0.05 ± 0.22 (0–1.0)	5.3
	*Moniezia*	6	48.6 ± 106.2 (0–400)	31.6
	*Capillaria*	0	na	na
	*Strongyloides*	0	na	na
	*Toxocara*	0	na	na
	*Eimeria*	2	0.28 ± 0.86 (0–3.6)	10.5
EINP plains bison herd (*n* = 23)	Strongyle	23	175.0 ± 143.2 (27.6–400)	100
	*Nematodirus*	6	1.3 ± 3.8 (0–18)	26.1
	*Trichuris*	0	na	na
	*Moniezia*	3	2.6 ± 8.4 (0–35.6)	13.0
	*Capillaria*	7	0.25 ± 0.70 (0–3.2)	30.4
	*Strongyloides*	0	na	na
	*Toxocara*	0	na	na
	*Eimeria*	16	55.7 ± 92.7 (0–400)	69.6
EINP wood bison herd (*n* = 16)	Strongyle	16	45.5 ± 37.8 (11.2–130.2)	100
	*Nematodirus*	4	0.76 ± 1.96 (0–7.2)	25
	*Trichuris*	3	0.06 ± 0.16 (0–0.6)	18.8
	*Moniezia*	8	26.6 ± 43.9 (0–133.3)	50
	*Capillaria*	4	0.19 ± 0.51 (0–2)	25
	*Strongyloides*	0	na	na
	*Toxocara*	0	na	na
	*Eimeria*	14	79.3 ± 97.1 (0–300)	87.5

*Abbreviations*: *GNP* Grasslands National Park, *EINP* Elk Island National Park, *n* number of individuals, *na* not applicable

In the case of the commercial bison herds, the overall mean strongyle, *Nematodirus****,*** *Trichuris* and *Moniezia* FECs were 72.0, 5.1, 18.0 and 21.3 epg, respectively. No significant differences were found in the strongyle (one-way ANOVA: *F*_(3, 54)_ = 0.174, *P* = 0.913), *Nematodirus* (one-way ANOVA: *F*_(3, 54)_ = 0.078, *P* = 0.972)*,* or *Moniezia* (one-way ANOVA: *F*_(3, 47)_ = 1.080, *P* = 0.367) mean FECs between provinces. However, *Trichuris* fecal egg counts were substantially higher in Saskatchewan (69.1 mean epg, one-way ANOVA: *F*_(3, 54)_ = 5.882, *P* = 0.002), Additional file 2: Table S1), compared to the other provinces, although this was no significance after *post-hoc* testing (Games-Howell *post-hoc*: AB *vs* SK *P* = 0.105; BC *vs* SK *P* = 0.105; MB *vs* SK *P* = 0.109). The average strongyle egg count of the cow-calf production groups from all provinces was 55.2 epg, while for the feeder production groups it was 101.8 epg. However, this difference was not statistically significant (independent samples t-test: *t*_(20.968)_ = -1.313, *P* = 0.203, equal variances not assumed). *Strongyloides* and *Toxocara* were not detected in any of the commercial herds and *Capillaria* was present in only one Alberta herd (34 epg). *Eimeria* oocysts were detected on all farms with a mean oocyst per gram (opg) of 656.7, with no significant differences between provinces or age groups (one-way ANOVA: *F*_(3, 47)_ = 1.833, *P* = 0.154).

There were significant differences in the mean strongyle fecal egg counts among all three conservation herds (one-way ANOVA: *F*_(2, 55)_ = 18.241, *P* < 0.0001, Games-Howell *post-hoc*, *P* < 0.007). The bison from the GNP plains bison herd had a mean strongyle egg count of 10.1 epg, while bison from the EINP plains and wood bison herds had mean strongyle egg counts of 175.0 and 45.5 epg, respectively. *Nematodirus* eggs were observed in the GNP bison herd (mean = 0.04 epg), the EINP plains herd (mean = 1.32 epg) and the EINP wood bison herd (mean = 0.76 epg). *Trichuris* eggs were only observed at a low level in the GNP herd (mean = 0.05 epg) and the EINP wood bison herd (mean = 0.06 epg), and were not observed in the EINP plains bison herd (Table 2). Neither *Strongyloides* nor *Toxocara* were detected in any of the conservation park herds. *Capillaria* was observed in 8 out of 23 bison sampled (mean = 0.25 epg) in the EINP plains bison herd, and in 3 out of 16 bison in the EINP wood bison herd (mean = 0.19 epg), but it was not detected in any animals from GNP. *Eimeria* was detected in GNP, EINP plains and EINP wood bison at 0.28 opg, 55.74 opg and 79.3 opg, respectively. *Moniezia* was detected in GNP (8/19 animals), EINP plains bison (3/23 animals) and EINP wood bison (8/16 animals) at a mean of 48.64 epg, 2.62 epg and 26.6 epg, respectively.

### Relative abundance of trichostrongylid nematode species in commercial and conservation bison herds determined by nemabiome metabarcoding

**Fig. 2:**
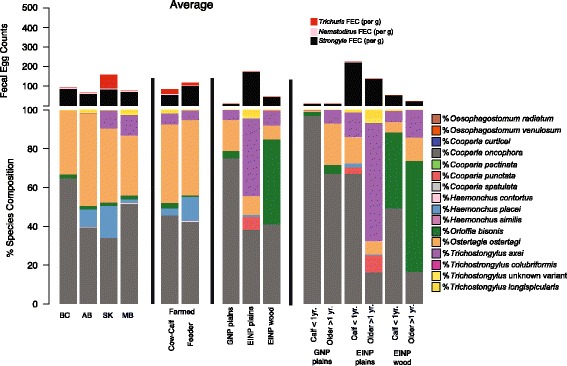
The mean nematode species proportions and egg counts by province, national park, production group and animal displayed in Fig. 1

Deep amplicon ITS2 rDNA nemabiome sequencing was performed on 1000–2000 larvae harvested from coprocultures to determine the individual species proportions of the different trichostrongylid nematode species that contribute to the strongyle egg count (Fig. 1). In the case of the commercial bison herds, *Cooperia oncophora* (43.1%) was the most abundant trichostrongylid nematode species overall, closely followed by *Ostertagia ostertagi* (39.9%), with these two species predominating in most herds (Figs. 1a, 2)*.* The next most abundant trichostrongylid nematode species overall was *Haemonchus placei*, with the overall percentages of 9.2% in Alberta, 16.6% in Saskatchewan, 1.7% in Manitoba, and 0.03% in BC (Fig. 2). However, the distribution of this parasite species was highly variable between farms, often being absent but being the predominant parasite in several herds (Fig. 1a). *Trichostrongylus axei* was similar in this regard being absent from most herds but being the predominant trichostrongyle species in one herd (two production groups) in Saskatchewan and two herds in Manitoba (Figs. 1a, 2). *Orloffia bisonis* was present at a low level in many herds, but was not the predominant parasite in any herd, and had an overall prevalence of ~2% in herds from all provinces (Fig. 1a).

The parasite species proportions were very similar in cow-calf production groups and feeder production groups. The only statistically significant difference was that cow-calf groups had a higher proportion of *Orloffia bisonis* (2.8%) compared to feeder groups (0.59%) (MetaStats: *P* = 0.02; Additional file 3: Table S2). The feeder groups also had a higher proportion of *H. placei* (12.5%) compared to cow-calf groups (3.6%); however, this was not significantly different (MetaStats: *P =* 0.14) (Additional file 3: Table S2).

The parasite communities present in the conservation herds were very different to those of the commercial bison herds and as well as to each other (Fig. 1b). The plains bison herd in GNP was the most similar to the commercial herds, with the most abundant trichostrongyle species being *C. oncophora* and *O. ostertagi* (75.0 and 16.0%, respectively) (Figs. 1b, 2). In contrast, the most abundant trichostrongyle species in EINP plains bison herd were *T. axei* (39.8%), *C. oncophora* (38.3%) and *O. ostertagi* (9.8%). It is noteworthy that *C. punctata* was present at 6.5% abundance in this herd but was not detected in any other herd in the study. Also of note was the difference between young (< 1 yr) and older animals (> 1 yr) in the EINP plains bison herd where *C. oncophora* (66.8%) and *T. axei* (60.9%) predominated, respectively (Fig. 2, Additional file 4: Table S3). The parasite community in the wood bison herd in EINP was the most different to all bison herds with abundant *Orloffia bisonis,* which was absent in the EINP plains bison herd, even though it was located in the same national park but in a separated fenced area (Figs. 1b, 2).

### Comparison of alpha diversity of trichostrongylid nematode communities by province and by national park

The alpha diversity, as measured by the inverse Simpson index, was determined for each commercial herd combined by province and each of the three national park herds: BC (1.8246); Alberta (1.9564); Saskatchewan (2.1277); Manitoba (2.0238); GNP plains bison herd (1.5179); EINP plains bison herd (1.9322); EINP wood bison herd (1.8564); all commercial herds (2.0003); all conservation herd samples (1.7841). There were no significant differences between observed alpha diversities (taking into account only number and evenness of species present) (one-way ANOVA: *F*_(6, 109)_ = 2.380, *P =* 0.034, Games-Howell *post-hoc* all comparisons: *P* > 0.069). However, when the populations were assessed with an AMOVA, all were significantly different (AMOVA: *P* < 0.001). This suggests that while the overall number and evenness of parasite species present is not significantly different between these populations, there were differences in the particular species present.

### Comparison of beta diversity of trichostrongylid nematode communities by province and by national park

Beta diversity, at the population level, among provinces and conservation herds was assessed with the weighted UniFrac function in Mothur, which revealed marked differences in the species present between populations (where a score of 0 indicates that populations are identical and a score of 1 indicates they are completely different) (Table 3). Overall, the EINP wood bison herd had the greatest difference in parasite community composition compared to the other herds (Table 3). The beta-diversity (UniFrac) between all commercial herds and all conservation herd populations was 0.481378, while the beta-diversity (UniFrac) between the cow-calf production groups compared to the feeder production groups for the commercial herds was 0.256381.

**Table 3:** Uni-Frac results. The Uni-Frac value assesses how similar the population structure of each of the populations is. A score of 0 indicates the populations are identical, while a score of 1 indicates they are entirely different. Pairwise comparisons are shown

	BC	AB	SK	MB	GNP-Plains	EINP-Plains	EINP-Wood
BC	–	0.655	0.628	0.301	0.401	0.583	0.697
AB	0.655	–	0.269	0.472	0.607	0.706	0.770
SK	0.628	0.269	–	0.449	0.583	0.617	0.807
MB	0.301	0.472	0.449	–	0.339	0.471	0.712
GNP-Plains	0.401	0.607	0.583	0.339	–	0.523	0.691
EINP-Plains	0.583	0.706	0.617	0.471	0.523	–	0.763
EINP-Wood	0.697	0.770	0.807	0.712	0.691	0.763	–

Beta diversity, at the species level, among populations was assessed with the MetaStats function in Mothur (pairwise *P*-values provided in Additional file 3: Table S2 and differences in species prevalence by animal age for the conservation herds is provided in Additional file 4: Table S3). While significant differences in species abundance were incorporated into the section in which the relative species abundance was presented, a complete breakdown of all observed significant differences can be found in Additional file 3: Table S2 and Additional file 4: Table S3).

### A novel *Trichostrongylus* ITS2 rDNA sequence variant from Canadian bison herds

**Fig. 3:**
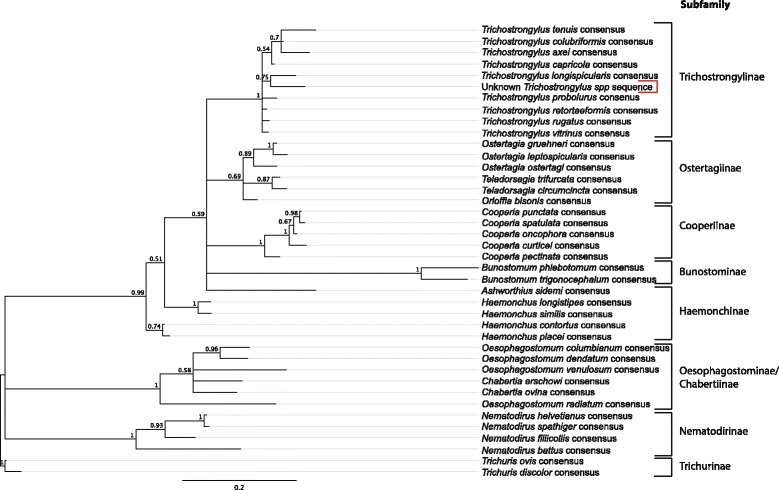
Phylogenetic tree of ITS2 consensus sequences from all species in analysis database. Consensus sequences for each *Trichostrongylus* spp. was generated from all available ITS2 sequences in GenBank (see Additional file 1: FASTA Database for sequences and accession numbers). The consensus sequence threshold was 75% and was generated using Geneious 10.1.3. The phylogenetic tree was computed using the MrBayes plugin with GTR substitution model, with 500,000 chain length, and rooted on the *Trichuris* consensus sequences (being Clade I nematodes compared to the other species which are primarily Clade V nematodes), using Geneious version 10.1.3 (created by Biomatters). Available from http://geneious.com/ [27]. Posterior probability values over 0.5 are shown, with values over 0.8 being considered significant. Sub-families are outlined, and the *Trichostrongylus* spp*.* sequence variant is highlighted with a red bracket

An ITS2 rDNA sequence was identified in both the conservation and commercial bison herds that was distinct from those of any *Trichostrongylus* species present in the public databases (Figs. 1a, b, 3, and Additional file 2: Table S1). Although this novel sequence had the highest level of identity to *T. vitrinus* (Additional file 5: Table S4), at 95.6% identity, it was phylogenetically closer to *Trichostrongylus longispicularis* sequences from the public databases despite only sharing 93.3% sequence identity (Fig. 3). Further, this sequence variant was always observed at a similar fixed ratio with *T. longispicularis* sequences in every population in which they were present (Fig. 1). This suggests that this novel sequence variant is an unreported ITS2 rDNA sequence variant of *T. longispicularis.* To confirm this, we directly sequenced ITS2 rDNA amplicons from 30 individual L3 larvae from a bison fecal sample by conventional Sanger sequencing. One of these larvae was identified as *T. longispicularis* by sequence identity, in addition to 20 *C. oncophora* and 9 *O. ostertagi.* The forward and reverse ITS2 rDNA sequence chromatograms from this *T. longispicularis* L3 was aligned to the two *T. longispicularis* sequences in the public databases (Accession Nos.: KY355070.1 and KY355068.1) and the novel *Trichostrongylus* sequence variant (Additional file 6: Figure S1). At each nucleotide position where the novel variant differs from the *T. longispicularis* reference sequences (highlighted), there is a double chromatogram peak corresponding to both sequence types. This confirms the novel sequence variant was derived from the same individual L3 larvae as contained the *T. longispicularis* ITS2 rDNA reference sequences.

## Discussion

Despite their clinical and economic importance, remarkably little is known about the prevalence and relative infection intensities of various parasite species in bison, and how these vary with geographical region or production system. Dies & Coupland (2001) conducted a fecal survey of 431 domestic bison from 22 herds to determine the prevalence of gastrointestinal helminth eggs [23]. Strongyle-type eggs were detected in 100% of samples, and *Capillaria* sp. in 63.6%, *Moniezia* sp*.* in 54.6%, *Nematodirus* sp*.* in 50%, *Trichuris* sp*.* in 40.9%, and *Strongyloides* sp. in 9.1% of fecal samples [23]. In 2014, a survey of parasite communities was undertaken on 98 farms in Manitoba and Saskatchewan to estimate the prevalence of *Toxocara vitulorum* in un-weaned bison calves [6]. That study estimated the prevalence of *T. vitulorum* at about 12% and 0% of herds in Saskatchewan and Manitoba, respectively. Herd-level prevalence for trichostrongyle eggs, *Eimeria* oocysts, *Monieza*, *Capillari*a, *Nematodirus*, *Trichuris* and *Strongyloides* eggs were estimated at 100%, 97%, 73%, 14%, 14%, 5% and 0% of herds, respectively [6]. However, the relative infection intensities of these parasites in each herd were not determined. A recent study used molecular techniques and PCR to identify genera of trichostrongyle in fecal samples taken from a large bison herd in South Dakota. Forty-eight of the 51 samples analyzed by PCR showed at least one trichostrongyle genera. *Ostertagia* sp. was demonstrated in 68.6% of the samples, *Cooperia* sp. in 80.39%, *Haemonchus* sp*.* in 73% and *Trichostrongylus* sp. in 16% of the 103 total samples taken from the herd. Bison were commonly infected with combinations of *Haemonchus*, *Ostertagia* and *Cooperia* [8].

### High trichostrongyle nematode parasite infection intensity and species diversity in bison relative to cattle herds in western Canada

We have previously studied and reported on gastro-intestinal trichostrongyle parasite communities in western Canadian beef cattle herds [14]. Although this study was not undertaken in parallel to the bison study, it was conducted in the same regions (British Columbia, Alberta, Saskatchewan and Manitoba) using a similar approach and so, it is interesting to compare the infection intensities and trichostrongyle species diversity found between the cattle and bison studies. The overall mean strongyle egg count for the 58 commercial bison production groups was 72.0 epg, compared to 12.5 epg for the 33 western Canadian beef cattle cow-calf herds sampled in 2012. This supports previous anecdotal opinion that commercial bison herds typically have much higher gastrointestinal parasite infection intensities than beef cattle. The nemabiome metabarcoding data suggests that there is also greater species diversity of trichostrongyle parasite communities in commercial bison herds than in beef cattle herds grazing similar pastures in western Canada [12].

Our study found the gastrointestinal trichostrongyle parasite communities of western Canadian beef cattle to be generally low, with *Ostertagia ostertagi* and *Cooperia oncophora* predominating in all herds and other parasite species rarely contributing more than > 1% of the parasite community [14]. There was a statistically significant difference in the overall parasite population diversity between the 58 western Canadian bison production groups (inverse Simpson Index: 2.006) studied here and the 33 western Canadian beef herds previously studied (inverse Simpson Index: 1.7410) (*t*-test: *t*_(88.773)_ = -3.359, *P =* 0.001, equal variances not assumed) [14]. This difference was also significant when assessed with an AMOVA (AMOVA: *P <* 0.001).

Although *O. ostertagi* and *C. oncophora* were also the most common trichostrongyle species in many commercial western Canadian bison herds, other trichostrongyle species were present at high levels, and indeed predominated in some herds. A number of the commercial bison herds had high levels of *Haemonchus placei* and this was the most abundant trichostrongyle species in five of the production groups surveyed (Fig. 1a). This is an important observation because this blood-feeding parasite is highly pathogenic and is not normally considered a major component of parasite communities at such northerly latitudes [14]. Although, *H. placei* is an important pathogen of cattle in the mid-western United States and further south, it is only present at very low levels in Canadian beef cattle herds (rarely more than 1% of the parasite community in a herd) [14]. The presence of *H. placei* at much higher levels in bison than in cattle grazing in the same climatic zone suggests bison are more permissive hosts for this parasite species. *Trichostrongylus axei* was the predominant species present in four of the 58 commercial bison production groups. This species is rarely reported as the predominant gastro-intestinal trichostrongyle species in livestock and it is unclear what would predispose this parasite to predominate in particular western Canadian bison herds. However, it again suggests the greater permissiveness of bison hosts compared to cattle for this parasite and underpins the importance of bison-specific investigations. The presence of *Orloffia bisonis* was of interest because this parasite, which is closely related to *O. ostertagi*, is considered to have co-evolved with bison, and is generally not found in domestic cattle. This is different to most of the other stronglyid parasites found in this study for which Bovidae are the primary natural hosts. *Orloffia bisonis* was detected in 18 of 58 production groups but was never the predominant species. There is limited information in regards to the pathogenicity of *O. bisonis*; however, it seems reasonable to assume it would have similar pathogenicity to *Ostertagia* spp. Nevertheless, these results suggest this bison parasite is likely to play a less important role in production and health impacts than the parasite species more commonly associated with cattle.

### Species composition of parasite communities in three conservation herds showed distinct differences to those of the commercial bison herds

We also investigated the gastrointestinal trichostrongyle parasite communities of three conservation national park bison herds. Elk Island National Park hosts the National Recovery herds for both plains bison and wood bison, which are kept in different containment areas in a virtually identical habitat, entirely separated by a highway and perimeter fencing. As both EINP and GNP herds are contained within entirely fenced areas (EINP plains bison: 136 km^2^; EINP wood bison: 64 km^2^; GNP: 181 km^2^), both parks currently manage their bison herds through biennial surplus to maintain the herd at pre-defined population targets (EINP) or carrying capacity (GNP: 400 ± 100 individuals). As per management guidelines for bison conservation herds [2], bison in both national parks are not typically treated with anthelmintics or vaccinated, unless major disease concerns arise. There were higher strongyle egg counts observed in the EINP plains bison (mean: 175.0 epg) compared to the GNP plains bison herd (mean: 11.2 epg).

There were some striking differences in the parasite communities of the conservation bison herds compared to the commercial bison herds, particularly for the EINP herds. Indeed, the parasite community structure differed more between the commercial and the conservation bison herds than between the commercial bison and cattle herds (weighted-UniFrac values of 0.481378 and 0.322076, respectively). The GNP herd was the most similar of the conservation herds to the commercial bison herds. In calves less than 1 year-old from the GNP herd, the parasite communities were almost exclusively *C. oncophora*, with *O. ostertagi* becoming increasingly predominant in older animals. Although our sample size is small, what is observed is similar to the situation commonly reported in cattle where acquired immunity is much more effective against *C. oncophora* leading to older cattle having much higher levels of *O. ostertagi* [24, 25]. In contrast, the parasite communities in the EINP plains bison herd were very different to the commercial herds. Although the major trichostrongyle parasite species present in calves < 1 year of age was also *C. oncophora*, *T. axei* overwhelmingly predominated in older animals in the EINP plains bison herd, comprising 60.9% of population (Fig. 2). In addition, *C. punctata* was seen at moderate levels in this herd even though it was not detected at all in any other commercial or conservation herd. The species profile of the EINP wood bison herd was notable by virtue of *Orloffia bisonis* being the most predominant trichostrongyle species present (Figs. 1b and 2). Interestingly*, O. bisonis* was either absent or only detected at very low levels in the commercial bison herds and the two plains bison conservation herds. This may reflect the different captive and ecological histories of the two bison types (wood and plains bison) present in EINP. Plains bison were introduced in 1907 directly from northern Montana. Wood bison also came from the southern part of the Northwest Territories. Since the two bison types have remained completely separated from each other and from local commercial cattle herds, they likely represent host-adapted parasites that were originally translocated with the founder animals and have persisted over time.

The predominance of *Ostertagia ostertagi* and *Haemonchus placei* in the commercial bison herds but not in the conservation herds is noteworthy. Because modern commercial bison herds largely graze on pastures previously used in cattle operations, it is likely that these two, primarily cattle parasite species, were originally acquired and maintained from pastures contaminated by cattle. It is possible that these parasite species have a higher pathogenicity in bison than in cattle since the former did not co-evolve with these parasites.

One caveat of this study to note is the propensity of *Ostertagia ostertag*i (and potentially *Orloffia bisonis*), to undergo hypobiosis in the winter months and sometimes re-emerge in the spring causing type-II ostertagiasis. Consequently, there could be significant numbers of inhibited *O. ostertagi* larvae present in some animals/herds. This would lead to an underestimation of the prevalence/infection intensities of *O. ostertagi* in this dataset as the inhibited larvae would not be reflected in the fecal egg count data.

### A novel *Trichostrongylus* ITS2 rDNA sequence present in bison is likely to be a divergent *T*. *longispicularis* sequence variant

An ITS2 rDNA sequence variant was identified in a number of the commercial and conservation herds for which there was no unambiguous sequence match in the public databases (Figs. 1 and 2). Although the highest overall sequence identity was with *T. vitrinus* (95.6% identity) sequences, it was most closely phylogenetically related to *T. longispicularis* (synonym *T. leroxi*) sequences (Additional file 6: Figure S1). It was considered possible that the sequence variant could be one of the ruminant *Trichostrongylus* spp. for which there are no available ITS2 rDNA reference sequences. These include *T. affinis*, *T. drepanoformis*, *T. dosteri*, *T. falculatus*, *T. hamatus*, *T. orientalis*, *T. skrjabini* and *T. askivali*. However, single worm sequencing determined the sequence variant to be present in the same individual L3 larva as those matching the publically available *T. longispicularis* sequences. This strongly suggests this is a *T. longispicularis* ITS2 rDNA sequence. If this is the case, the intra-species sequence identity of 93.3% is unusually low, as intra-species sequence identity is generally 97% or higher in trichostronglyid nematodes due to concerted evolution. It is possible that the sequenced individual larva was the progeny of hybridization of two closely related *Trichostrongylus* species as has been shown to occur in the genus *Haemonchus* [26]. However, this seems highly unlikely given the fixed ratio between the novel sequence variant and the *T. longispicularis* reference sequences in all populations where either one is present. This latter observation is much more consistent with the novel sequence variant being present in the same polycistronic array as the *T. longispicularis* reference sequences. Overall, this demonstrates the need for ITS2 rDNA databases with morphologically validated species and more representation to capture the full extent of sequence variation and enable reliable species assignment.

## Conclusions

The trichostrongyle group of gastrointestinal nematode parasites are an important cause of clinical disease in North American bison and of significance for both the commercial farming industry and conservation efforts. The group of parasites in this study includes many different species that cannot be distinguished by visual microscopic examination of eggs present in feces. Consequently, we recently developed a new approach to quantify the species composition of trichostrongyle nematode communities in cattle based on deep amplicon sequencing of the ITS2 rDNA locus in populations of L3 larvae harvested from fecal cultures. Here, we show the power of this approach applied to bison herds. It has allowed us to screen a large number of bison herds to quantify the parasite trichostrongyle species present on a scale that would not have been possible using traditional morphological techniques. The dataset presented in this paper represents the most comprehensive assessment of trichostrongyle infecting bison to date, by far. We report that the predominant trichostrongyle parasite species in commercial western Canadian bison herds are those normally associated with cattle; however, they are present at higher infection intensities and higher parasite species diversity than occurs in beef cattle grazing at the same latitude. *Ostertagia ostertagi* is the predominant species overall, which combined with high fecal egg counts in many herds, supports the view that there is a significant risk of clinical disease and production impacts associated with gastro-intestinal nematode parasites in western Canadian bison herds. Of particular note was the fact that *H. placei* was also present at high levels in some herds, which is of significant concern as this is a highly pathogenic parasite. This trichostrongyle species is not considered likely to thrive at such northerly latitudes and is only present at very low level in cattle parasite communities in Canada or the northern United States. Finally, we have found that the trichostrongyle parasite communities in bison herds from several conservation herds differ notably from that in commercial bison herds, including the presence of *O. bisonis* in the wood bison conservation herd.

## Additional files

Additional file 1: FASTA Database. (FASTA 797 kb)

Additional file 2: **Table S1.** Fecal egg counts by province, herd and production group. (DOCX 98 kb)

Additional file 3: **Table S2.** MetaStats Results by species. (DOCX 157 kb)

Additional file 4: **Table S3.** MetaStats results for younger versus older animals from conservation herds. (DOCX 53 kb)

Additional file 5: **Table S4.** Pairwise percent identity between *Trichostrongylus* spp*. (DOCX 70 kb)*

Additional file 6: **Figure S1.** Alignment of *Trichostrongylus longispicularis* ITS2 sequences. A single *Trichostrongylus longispicularis* L3 larvae was identified, and the ITS2 region was amplified by PCR. The PCR product was sent for conventional Sanger sequencing in the forward and reverse directions. The chromatogram for the forward and reverse sequences, along with the two available *T. longispicularis* ITS2 sequences (KY355070.1 and KY355068.1) and the unknown *Trichostrongylus* sequence variant were aligned. Sequences were aligned with the MUSCLE alignment using default parameters [28]. (PDF 554 kb)

## Abbreviations

AB: Alberta
AMOVA: Analysis of Molecular Variance
ANOVA: Analysis of Variance
BC: British Columbia
DNA: deoxyribonucleic acid
EINP: Elk Island National Park
EPG: eggs per gram
FEC: fecal egg count
GNP: Grasslands National Park
ITS2: internal transcribed spacer 2
L3: third-stage larvae
MB: Manitoba
OPG: oocyst per gram
PCR: polymerase chain reaction
rDNA: ribosomal DNA
SK: Saskatchewan

## References

[1] Freese CH, Aune KE, Boyd DP, Derr JN, Forrest SC, Cormack Gates C (2007). Second chance for the plains bison. Biol Conservation..

[2] Gates CC, Freese CH, Gogan PJP, Kotzman M. American bison status survey and conservation guidelines 2010. 2010. papers3://publication/uuid/DE9E6459-40F7-48E3-8CA4-63834210C978.

[3] Boyd DP (2003). Conservation of North American bison: status and recommendations. PhD thesis.

[4] Carter D, Matheson J. Bullish on bison: an island of stability and profitability in today’s agricultural economy. 2017. https://bisoncentral.com/wp-content/uploads/2016/12/BisonMarketOverview_Bullish_10_17.pdf. Accessed 20 Oct 2017.

[5] National Bison Association. Advantages of bison: deliciously healthy bison. 2017. https://bisoncentral.com/advantage-item/deliciously-healthy-bison/. Accessed 20 Oct 2017.

[6] Woodbury MR, Wagner B, Ben-ezra E, Douma D, Wilkins W (2014). A survey to detect *Toxocara vitulorum* and other gastrointestinal parasites in bison (*Bison bison*) herds from Manitoba and Saskatchewan. Can Vet J.

[7] Wade SE, Haschek WM, Georgi JR (1979). Ostertagiosis in captive bison in New York State: report of nine cases. Cornell Vet.

[8] Eljaki AA, Al Kappany YM, Grosz DD, Smart AJ, Hildreth MB (2016). Molecular survey of trichostrongyle nematodes in a *Bison bison* herd experiencing clinical parasitism, and effects of avermectin treatment. Vet Parasitol.

[9] Pelletier F, Page KA, Ostiguy T, Festa-Bianchet M (2005). Fecal counts of lungworm larvae and reproductive effort in bighorn sheep, *Ovis canadensis*. Oikos.

[10] Gulland FMD (1992). The role of nematode parasites in Soay sheep (*Ovis aries* L.) mortality during a population crash. Parasitology.

[11] Hoberg EP, Kocan AA, Rickard LG, William SM, Pybus MJ, Kocan AA (2001). Gastrointestinal strongyles in wild ruminants. Parasitic diseases of wild mammals. Ames, Iowa.

[12] Marley SE, Knapp SE, Rognlie MC, Thompson JR, Stoppa TM, Button SM (1995). Efficacy of ivermectin pour-on against *Ostertagia ostertagi* infection and residues in the American bison, *Bison bison*. J Wildl Dis.

[13] Avramenko RW, Redman EM, Lewis R, Yazwinski TA, Wasmuth JD, Gilleard JS (2015). Exploring the gastrointestinal “nemabiome”: deep amplicon sequencing to quantify the species composition of parasitic nematode communities. PLoS One.

[14] Avramenko RW, Redman EM, Lewis R, Bichuette MA, Palmeira BM, Yazwinski TA (2017). The use of nemabiome metabarcoding to explore gastro-intestinal nematode species diversity and anthelmintic treatment effectiveness in beef calves. Int J Parasitol.

[15] Bras AL, Barkema HW, Woodbury M, Ribble C, Perez-Casal J, Windeyer MC (2016). Risk factors for *Mycoplasma bovis*-associated disease in farmed bison (*Bison bison*) herds in western Canada: a case-control study. Prev Vet Med.

[16] Cox P, Todd A (1962). Survey of gastrointestinal parasitism in Wisconsin. J Am Vet Med Assoc.

[17] Roberts F, O’Sullivan P (1950). Methods for egg counts and larval cultures for strongyles infesting the gastro-intestinal tract of cattle. Aust J Agric Res.

[18] Schloss PD, Westcott SL, Ryabin T, Hall JR, Hartmann M, Hollister EB (2009). Introducing mothur: open-source, platform-independent, community-supported software for describing and comparing microbial communities. Appl Environ Microbiol.

[19] Simpson EH (1949). Measurement of diversity. Nature.

[20] Lozupone C, Knight R (2005). UniFrac: a new phylogenetic method for comparing microbial communities. Appl Environ Microbiol.

[21] Bray JR, Curtis JT (1957). An ordination of the upland forest communities of southern Wisconsin. Ecol Monogr..

[22] White JR, Nagarajan N, Pop M (2009). Statistical methods for detecting differentially abundant features in clinical metagenomic samples. PLoS Comput Biol.

[23] Dies KH, Coupland RW (2001). Prevalence of gastrointestinal helminths in domestic bison herds in northwestern Alberta. Can Vet J.

[24] Gasbarre LC, Leighton EA, Davies CJ (1990). Genetic control of immunity to gastrointestinal nematodes of cattle. Vet Parasitol.

[25] Gasbarre LC, Leighton EA, Sonstegard T (2001). Role of the bovine immune system and genome in resistance to gastrointestinal nematodes. Vet Parasitol.

[26] Chaudhry U, Redman EM, Abbas M, Muthusamy R, Ashraf K, Gilleard JS (2015). Genetic evidence for hybridisation between *Haemonchus contortus* and *Haemonchus placei* in natural field populations and its implications for interspecies transmission of anthelmintic resistance. Int J Parasitol.

[27] Huelsenbeck JP, MrBayes RF (2001). Bayesian inference of phylogeny. Bioinformatics.

[28] Edgar RC (2010). Search and clustering orders of magnitude faster than BLAST. Bioinformatics..

