# Supplementary material for: High species diversity of trichostrongyle parasite communities within and between Western Canadian commercial and conservation bison herds revealed by nemabiome metabarcoding
Source: Parasit Vectors. 2018 May 15;11:299. doi: 10.1186/s13071-018-2880-y (PMC5952520; doi:10.1186/s13071-018-2880-y)
Supplement: Supplementary file 2 — Table S1. Fecal egg counts by province, herd and production group. (DOCX 98 kb) [file 13071_2018_2880_MOESM2_ESM.docx]

**Additional file 2: Table S1.**

Fecal egg counts by province, herd and production group

|  | Province | Mean | Std. Dev | Std. Error | 95% CI Lower | 95% CI Higher |
| --- | --- | --- | --- | --- | --- | --- |
| *Strongyle* | BC | 86.70 | 107.25 | 47.96 | -46.47 | 219.57 |
|  | AB | 61.96 | 103.13 | 21.05 | 18.41 | 105.51 |
|  | SK | 83.30 | 121.16 | 31.28 | 16.21 | 150.39 |
|  | MB | 71.64 | 69.99 | 18.71 | 31.23 | 112.05 |
|  | GNP – Plains | 10.11 | 9.23 | 2.12 | 5.65 | 14.56 |
|  | EINP – Plains | 175.1 | 143.22 | 29.86 | 113.08 | 236.94 |
|  | EINP – Wood | 45.49 | 37.77 | 9.44 | 25.36 | 65.61 |
|  | Cow-Calf group (Commercial) | 55.23 | 58.31 | 9.72 | 35.51 | 74.97 |
|  | Feeder group (Commercial) | 101.82 | 148.72 | 34.12 | 30.14 | 173.50 |
| *Nematodirus* | BC | 6.80 | 15.21 | 6.80 | -12.08 | 25.68 |
|  | AB | 4.96 | 12.51 | 2.55 | -0.32 | 10.25 |
|  | SK | 5.67 | 9.46 | 2.44 | 0.43 | 10.90 |
|  | MB | 4.25 | 9.28 | 2.48 | -1.11 | 9.61 |
|  | GNP – Plains | 0.04 | 0.08 | 0.02 | 0.00 | 0.08 |
|  | EINP – Plains | 1.32 | 3.81 | 0.79 | -0.33 | 2.97 |
|  | EINP – Wood | 0.76 | 1.96 | 0.49 | 0.04 | 1.92 |
|  | Cow-Calf group (Commercial) | 7.08 | 13.09 | 2.18 | 2.65 | 11.51 |
|  | Feeder group (Commercial) | 2.24 | 5.55 | 1.27 | 0 | 17.00 |
| *Trichuris* | BC | 0 | 0 | 0 | 0 | 0 |
|  | AB | 0 | 0 | 0 | 0 | 0 |
|  | SK | 69.13 | 107.46 | 27.75 | 9.62 | 128.64 |
|  | MB | 0.61 | 2.27 | 0.61 | -0.70 | 1.92 |
|  | GNP – Plains | 0.05 | 0.23 | 0.05 | -0.06 | 0.16 |
|  | EINP – Plains | 0 | 0 | 0 | 0 | 0 |
|  | EINP – Wood | 0.06 | 0.16 | 0.04 | 0.00 | 0.16 |
|  | Cow-Calf group (Commercial) | 21.49 | 74.59 | 12.43 | -3.75 | 46.72 |
|  | Feeder group (Commercial) | 14.32 | 31.81 | 7.30 | -1.02 | 29.65 |
| *Eimeria* | BC | 571.20 | 421.00 | 188.28 | 42.68 | 495.32 |
|  | AB | 269.00 | 440.19 | 106.76 | 42.68 | 495.32 |
|  | SK | 1172.43 | 1915.18 | 494.50 | 111.84 | 2233.03 |
|  | MB | 605.32 | 329.88 | 88.1 | 414.86 | 795.79 |
|  | GNP – Plains | 0.28 | 0.86 | 0.19 | 0.00 | 0.71 |
|  | EINP – Plains | 55.74 | 92.87 | 19.87 | 24.67 | 98.47 |
|  | EINP – Wood | 79.33 | 97.07 | 24.27 | 35.08 | 133.02 |
|  | Cow-Calf group (Commercial) | 626.50 | 1226.78 | 210.40 | 198.46 | 1054.54 |
|  | Feeder group (Commercial) | 717.00 | 916.38 | 222.25 | 245.84 | 1188.16 |
| *Moniezia* | BC | 44.20 | 98.83 | 44.20 | -78.52 | 166.92 |
|  | AB | 35.00 | 77.55 | 18.81 | -4.87 | 74.87 |
|  | SK | 6.80 | 17.95 | 4.63 | -3.14 | 16.74 |
|  | MB | 12.14 | 36.17 | 9.67 | -8.74 | 33.03 |
|  | GNP – Plains | 48.64 | 106.19 | 23.47 | 9.69 | 105.14 |
|  | EINP – Plains | 2.62 | 8.43 | 1.73 | 0.00 | 6.59 |
|  | EINP – Wood | 26.61 | 43.87 | 10.97 | 6.60 | 51.80 |
|  | Cow-Calf group (Commercial) | 22.50 | 48.94 | 8.39 | 5.43 | 39.57 |
|  | Feeder group (Commercial) | 19.00 | 74.07 | 17.97 | -19.08 | 57.08 |
