# Supplementary material for: High species diversity of trichostrongyle parasite communities within and between Western Canadian commercial and conservation bison herds revealed by nemabiome metabarcoding
Source: Parasit Vectors. 2018 May 15;11:299. doi: 10.1186/s13071-018-2880-y (PMC5952520; doi:10.1186/s13071-018-2880-y)
Supplement: Supplementary file 3 — Table S2. MetaStats Results by species. (DOCX 157 kb) [file 13071_2018_2880_MOESM3_ESM.docx]

**Additional file 3: Table S2.**

MetaStats Results by species

| ***C. oncophora*** |  | **BC^a^** | **AB^a^** | **SK^a^** | **MB^a^** | **GNP-Plains^b^** | **EINP-Plains^b^** | **EINP-Wood^b^** | **Cow-Calf ^a^** | **Feeder ^a^** |
| --- | --- | --- | --- | --- | --- | --- | --- | --- | --- | --- |
|  | **BC^a^** | / | 0.029* | 0.009* | 0.273 | 0.588 | 0.068 | 0.062 | / | / |
|  | **AB^a^** | 0.029* | / | 0.474 | 0.146 | 0.003* | 0.965 | 0.949 | / | / |
|  | **SK^a^** | 0.009* | 0.474 | / | 0.051 | 0.002* | 0.563 | 0.583 | / | / |
|  | **MB^a^** | 0.273 | 0.146 | 0.051 | / | 0.015* | 0.226 | 0.223 | / | / |
|  | **GNP-Plains^b^** | 0.588 | 0.003* | 0.002* | 0.015* | / | 0.006* | 0.001 | / | / |
|  | **EINP-Plains^b^** | 0.068 | 0.965 | 0.563 | 0.226 | 0.006* | / | 0.990 | / | / |
|  | **EINP-Wood^b^** | 0.062 | 0.949 | 0.583 | 0.223 | 0.001* | 0.990 | / | / | / |
|  | **Cow-Calf ^a^** | / | / | / | / | / | / | / | / | 0.682 |
|  | **Feeder^a^** | / | / | / | / | / | / | / | 0.682 | / |
| ***O. ostertagi*** |  | **BC^a^** | **AB^a^** | **SK^a^** | **MB^a^** | **GNP-Plains^b^** | **EINP-Plains^b^** | **EINP-Wood^b^** | **Cow-Calf ^a^** | **Feeder ^a^** |
|  | **BC^a^** | / | 0.170 | 0.644 | 0.835 | 0.112 | 0.0560 | 0.021* | / | / |
|  | **AB^a^** | 0.170 | / | 0.247 | 0.031* | 0.003* | 0.001* | 0.001* | / | / |
|  | **SK^a^** | 0.644 | 0.247 | / | 0.409 | 0.028* | 0.001* | 0.001* | / | / |
|  | **MB^a^** | 0.835 | 0.031* | 0.409 | / | 0.078 | 0.001* | 0.001* | / | / |
|  | **GNP-Plains^b^** | 0.112 | 0.003* | 0.028* | 0.078 | / | 0.412 | 0.141 | / | / |
|  | **EINP-Plains^b^** | 0.0560 | 0.001* | 0.001* | 0.001* | 0.412 | / | 0.301 | / | / |
|  | **EINP-Wood^b^** | 0.021* | 0.001* | 0.001* | 0.001* | 0.141 | 0.301 | / | / | / |
|  | **Cow-Calf ^a^** | / | / | / | / | / | / | / | / | 0.833 |
|  | **Feeder^a^** | / | / | / | / | / | / | / | 0.833 | / |
| ***T. axei*** |  | **BC^a^** | **AB^a^** | **SK^a^** | **MB^a^** | **GNP-Plains^b^** | **EINP-Plains^b^** | **EINP-Wood^b^** | **Cow-Calf ^a^** | **Feeder ^a^** |
|  | **BC^a^** | / | 0.295 | 0.146 | 0.202 | 0.013* | 0.000* | 0.002* | / | / |
|  | **AB^a^** | 0.295 | / | 0.219 | 0.339 | 0.011* | 0.001* | 0.001* | / | / |
|  | **SK^a^** | 0.146 | 0.219 | / | 0.818 | 0.537 | 0.007* | 0.720 | / | / |
|  | **MB^a^** | 0.202 | 0.339 | 0.818 | / | 0.554 | 0.001* | 0.693 | / | / |
|  | **GNP-Plains^b^** | 0.013* | 0.011* | 0.537 | 0.554 | / | 0.001* | 0.501 | / | / |
|  | **EINP-Plains^b^** | 0.000* | 0.001* | 0.007* | 0.001* | 0.001* | / | 0.001* | / | / |
|  | **EINP-Wood^b^** | 0.002* | 0.001* | 0.720 | 0.693 | 0.501 | 0.001* | / | / | / |
|  | **Cow-Calf ^a^** | / | / | / | / | / | / | / | / | 0.779 |
|  | **Feeder^a^** | / | / | / | / | / | / | / | 0.779 | / |
| ***O. bisonis*** |  | **BC^a^** | **AB^a^** | **SK^a^** | **MB^a^** | **GNP-Plains^b^** | **EINP-Plains^b^** | **EINP-Wood^b^** | **Cow-Calf ^a^** | **Feeder^a^** |
|  | **BC^a^** | / | 0.848 | 0.794 | 0.863 | 0.624 | 0.369 | 0.000* | / | / |
|  | **AB^a^** | 0.848 | / | 0.957 | 0.936 | 0.224 | 0.003* | 0.001* | / | / |
|  | **SK^a^** | 0.794 | 0.957 | / | 0.961 | 0.237 | 0.001* | 0.001* | / | / |
|  | **MB^a^** | 0.863 | 0.936 | 0.961 | / | 0.370 | 0.004* | 0.001* | / | / |
|  | **GNP-Plains^b^** | 0.624 | 0.224 | 0.237 | 0.370 | / | 0.001* | 0.001* | / | / |
|  | **EINP-Plains^b^** | 0.369 | 0.003* | 0.001* | 0.004* | 0.001* | / | 0.001* | / | / |
|  | **EINP-Wood^b^** | 0.000* | 0.001* | 0.001* | 0.001* | 0.001* | 0.001* | / | / | / |
|  | **Cow-Calf ^a^** | / | / | / | / | / | / | / | / | 0.026* |
|  | **Feeder^a^** | / | / | / | / | / | / | / | 0.026* | / |
| ***H. placei*** |  | **BC^a^** | **AB^a^** | **SK^a^** | **MB^a^** | **GNP-Plains^b^** | **EINP-Plains^b^** | **EINP-Wood^b^** | **Cow-Calf ^a^** | **Feeder^a^** |
|  | **BC^a^** | / | 0.059 | 0.006* | 0.030* | 0.139 | 0.232 | 0.270 | / | / |
|  | **AB^a^** | 0.059 | / | 0.295 | 0.136 | 0.05* | 0.079 | 0.009* | / | / |
|  | **SK^a^** | 0.006* | 0.295 | / | 0.011* | 0.001* | 0.011* | 0.001* | / | / |
|  | **MB^a^** | 0.030* | 0.136 | 0.012* | / | 0.001* | 0.813 | 0.003* | / | / |
|  | **GNP-Plains^b^** | 0.139 | 0.005* | 0.001* | 0.001* | / | 0.005* | 0.209 | / | / |
|  | **EINP-Plains^b^** | 0.232 | 0.079 | 0.011* | 0.813 | 0.005* | / | 0.013* | / | / |
|  | **EINP-Wood^b^** | 0.270 | 0.009* | 0.001* | 0.003* | 0.209 | 0.013* | / | / | / |
|  | **Cow-Calf ^a^** | / | / | / | / | / | / | / | / | 0.140 |
|  | **Feeder^a^** | / | / | / | / | / | / | / | 0.140 | / |
| ***T. longispicularis*** |  | **BC^a^** | **AB^a^** | **SK^a^** | **MB^a^** | **GNP-Plains^b^** | **EINP-Plains^b^** | **EINP-Wood^b^** | **Cow-Calf ^a^** | **Feeder ^a^** |
|  | **BC^a^** | / | 0.124 | 0.726 | 0.265 | 0.350 | 0.018* | 0.724 | / | / |
|  | **AB^a^** | 0.124 | / | 0.155 | 0.570 | 0.001* | 0.053 | 0.225 | / | / |
|  | **SK^a^** | 0.726 | 0.155 | / | 0.432 | 0.001* | 0.002* | 0.991 | / | / |
|  | **MB^a^** | 0.265 | 0.570 | 0.432 | / | 0.003* | 0.384 | 0.235 | / | / |
|  | **GNP-Plains^b^** | 0.350 | 0.001* | 0.001* | 0.003* | / | 0.001* | 0.001* | / | / |
|  | **EINP-Plains^b^** | 0.018* | 0.053 | 0.002* | 0.384 | 0.001* | / | 0.001* | / | / |
|  | **EINP-Wood^b^** | 0.724 | 0.225 | 0.991 | 0.235 | 0.001* | 0.001* | / | / | / |
|  | **Cow-calf^a^** | / | / | / | / | / | / | / | / | 0.117 |
|  | **Feeder ^a^** | / | / | / | / | / | / | / | 0.117 | / |
| ***C. punctata*** |  | **BC^a^** | **AB^a^** | **SK^a^** | **MB^a^** | **GNP-Plains^b^** | **EINP-Plains^b^** | **EINP-Wood^b^** | **Cow-Calf ^a^** | **Feeder ^a^** |
|  | **BC^a^** | / | 1.000 | 0.578 | 1.000 | 1.000 | 0.122 | 1.000 | / | / |
|  | **AB^a^** | 1.000 | / | 0.162 | 1.000 | 1.000 | 0.004* | 1.000 | / | / |
|  | **SK^a^** | 0.578 | 0.162 | / | 0.251 | 0.086 | 0.066 | 0.113 | / | / |
|  | **MB^a^** | 1.000 | 1.000 | 0.251 | / | 1.000 | 0.026* | 1.000 | / | / |
|  | **GNP-Plains^b^** | 1.000 | 1.000 | 0.086 | 1.000 | / | 0.034* | 1.000 | / | / |
|  | **EINP-Plains^b^** | 0.122 | 0.0040* | 0.066 | 0.026* | 0.034* | / | 0.027 | / | / |
|  | **EINP-Wood^b^** | 1.000 | 1.000 | 0.113 | 1.000 | 1.000 | 0.027 | / | / | / |
|  | **Cow-Calf ^a^** | / | / | / | / | / | / | / | / | 0.556 |
|  | **Feeder^a^** | / | / | / | / | / | / | / | 0.556 | / |

Commercial herds^a^, Conservation herds^b^. *P* < 0.05*
