# Supplementary material for: High species diversity of trichostrongyle parasite communities within and between Western Canadian commercial and conservation bison herds revealed by nemabiome metabarcoding
Source: Parasit Vectors. 2018 May 15;11:299. doi: 10.1186/s13071-018-2880-y (PMC5952520; doi:10.1186/s13071-018-2880-y)
Supplement: Supplementary file 4 — Table S3. MetaStats results for younger versus older animals from conservation herds. (DOCX 53 kb) [file 13071_2018_2880_MOESM4_ESM.docx]

**Additional file 4: Table S3.**

MetaStats results for younger versus older animals from conservation herds

|  | **GNP Plains**  **Younger (<1yr) vs Older (>1yr.)** | | | **EINP Island Plains**  **Younger (<1yr) vs Older (>1yr.)** | | | **EINP Wood**  **Younger (<1yr) vs Older (>1yr.)** | | |
| --- | --- | --- | --- | --- | --- | --- | --- | --- | --- |
|  | Young (mean) | Older (mean) | *P* value | Young (mean) | Older (mean) | *P* value | Young (mean) | Older (mean) | *P* value |
| *C. oncophora* | 96.98 | 67.15 | 0.024 | 66.83 | 16.34 | 0.001 | 49.33 | 16.68 | 0.099 |
| *O. ostertagi* | 0.93 | 21.40 | 0.022 | 13.57 | 6.92 | 0.305 | 5.45 | 12.04 | 0.054 |
| *T. axei* | 0.01 | 6.95 | 0.004 | 12.53 | 60.87 | 0.001 | 5.60 | 14.11 | 0.096 |
| *O. bisonis* | 2.04 | 4.48 | 0.331 | 0 | 0 | 1 | 39.24 | 57.17 | 0.148 |
| *H. placei* | 0 | 0 | 1 | 2.05 | 0.48 | 0.567 | 0 | 0 | 1 |
| *T. longispicularis* | 0 | 0 | 1 | 1.29 | 6.57 | 0.028 | 0.38 | 0 | 0.202 |
| *C. punctata* | 0 | 0 | 1 | 3.52 | 8.81 | 0.437 | 0 | 0 | 1 |
