# Supplementary material for: High species diversity of trichostrongyle parasite communities within and between Western Canadian commercial and conservation bison herds revealed by nemabiome metabarcoding
Source: Parasit Vectors. 2018 May 15;11:299. doi: 10.1186/s13071-018-2880-y (PMC5952520; doi:10.1186/s13071-018-2880-y)
Supplement: Supplementary file 5 — Table S4. Pairwise percent identity between Trichostrongylus spp. (DOCX 70 kb) [file 13071_2018_2880_MOESM5_ESM.docx]

**Additional file 5: Table S4.**

Pairwise Percent Identity Between *Trichostrongylus spp.*

|  | **Tt** | **Tb** | **Tl** | **TSV** | **Tr** | **Trug** | **Tca** | **Tco** | **Tv** | **Ta** |
| --- | --- | --- | --- | --- | --- | --- | --- | --- | --- | --- |
| **Tt** | 90.5 | 91.2 | 91.6 | 92.2 | 92.4 | 93.1 | 93.1 | 93.5 | 92 | 92 |
| **Tp** | 90.5 | / | 91.8 | 91.8 | 93.8 | 94.4 | 94.5 | 93.8 | 95 | 93.5 |
| **Tl** | 91.2 | 91.8 | / | 93.3 | 92.9 | 93.5 | 94.7 | 94.2 | 94.7 | 92.7 |
| **TSV** | 91.6 | 91.8 | 93.3 | / | 93.3 | 93.5 | 94.7 | 94.2 | 95.6 | 93.1 |
| **Tr** | 92.2 | 93.8 | 92.9 | 93.3 | / | 97.3 | 96.4 | 96.7 | 96.6 | 96.9 |
| **Trug** | 92.4 | 94.4 | 93.5 | 93.5 | 97.3 | / | 96.4 | 96.7 | 96.8 | 96.3 |
| **Tca** | 93.1 | 94.5 | 94.7 | 94.7 | 96.4 | 96.4 | / | 99.6 | 96.6 | 96.4 |
| **Tco** | 93.1 | 93.8 | 94.2 | 94.2 | 96.7 | 96.7 | 99.6 | / | 96.6 | 96 |
| **Tv** | 93.5 | 95 | 94.7 | 95.6 | 96.6 | 96.8 | 96.6 | 96.6 | / | 96.4 |
| **Ta** | 92 | 93.5 | 92.7 | 93.1 | 96.9 | 96.3 | 96.4 | 96 | 96.4 | / |

Tt = *T. tenuis;* Tp *= T. probolurus*; Tl = *T. longispicularis*; TSV = *Trichostrongylus* Sequence Variant; Tr = *T. retortaeformis*; Trug = *T. rugatus*; Tca = *T. capricola*; Tco = *T. colubriformis*; Tv = *T. vitrinus*; Ta = *T. axei*.
