# Supplementary figures and images for: High species diversity of trichostrongyle parasite communities within and between Western Canadian commercial and conservation bison herds revealed by nemabiome metabarcoding
Source: Parasit Vectors. 2018 May 15;11:299. doi: 10.1186/s13071-018-2880-y (PMC5952520; doi:10.1186/s13071-018-2880-y)

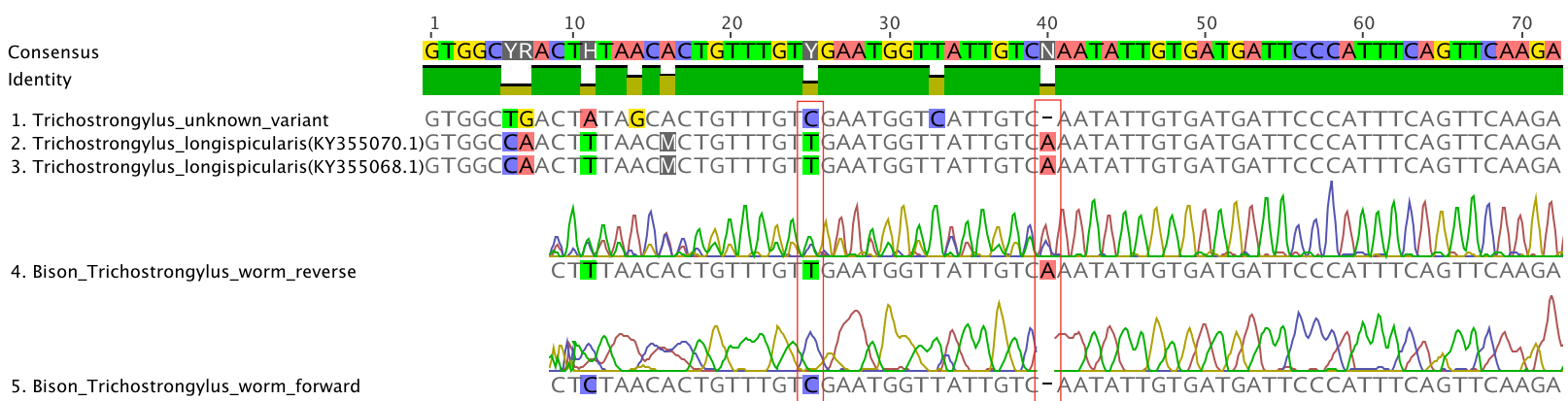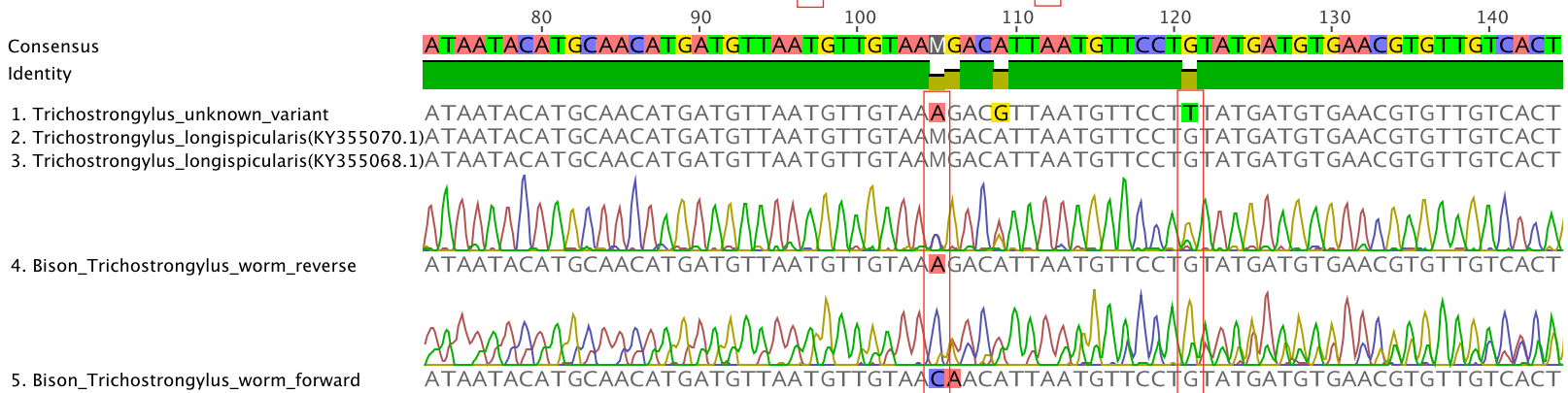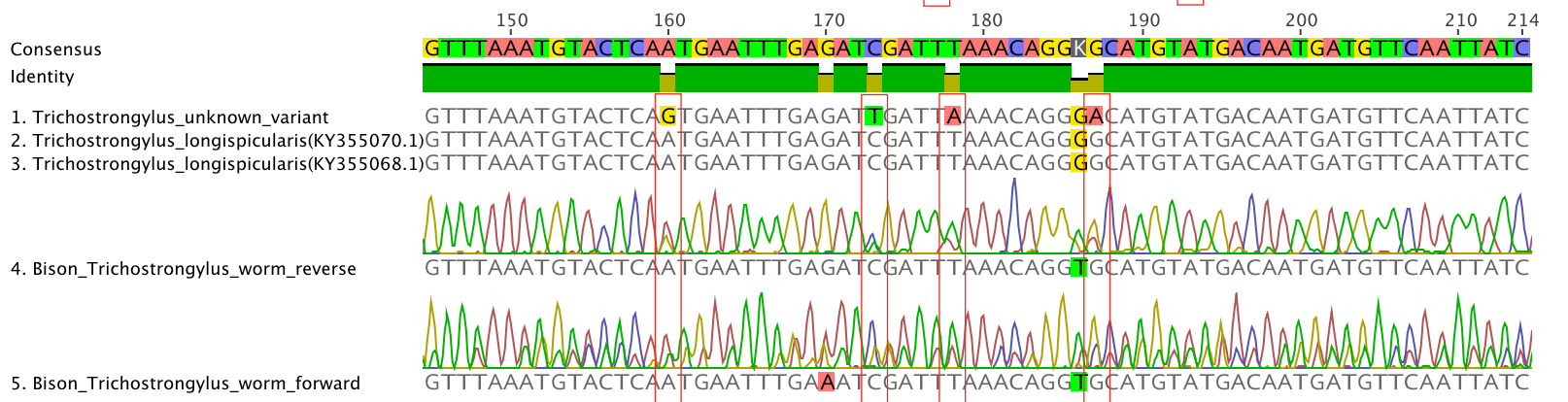

Supplement: Supplementary file 6 — Figure S1. Alignment of Trichostrongylus longispicularis ITS2 sequences. A single Trichostrongylus longispicularis L3 larvae was identified, and the ITS2 region was amplified by PCR. The PCR product was sent for conventional Sanger sequencing in the forward and reverse directions. The chromatogram for the forward and reverse sequences, along with the two available T. longispicularis ITS2 sequences (KY355070.1 and KY355068.1) and the unknown Trichostrongylus sequence variant were aligned. Sequences were aligned with the MUSCLE alignment using default parameters [28]. (PDF 554 kb) [file 13071_2018_2880_MOESM6_ESM.pdf]
